# Identification of marker allergens in Brazilian soy allergic patients

**DOI:** 10.1186/2045-7022-1-S1-P72

**Published:** 2011-08-12

**Authors:** Renata Cocco, Dirceu Solé, Marcia Mallozi, Charles Naspitz, Fredrik Bernhardsson, Sigrid Sjolander, Maryam Poorafshar, Anita Kober

**Affiliations:** 1 Federal University of São Paulo, Pediatrics, São Paulo, Brazil; 2 Phadia AB, Uppsala, Sweden

## Background

Soy allergy is established as one of the most common food allergies. Therefore, we decided to analyze whether there are specific protein markers among Brazilian soy allergic patients, using microarray techniques.

## Method

Thirteen patients presenting with immediate symptoms after ingestion of soy formula and IgE reactivity to soy extract were selected after failing oral food challenges. Sixteen patients with no symptoms (negative soy challenge), but presenting with positive specific IgE to soy (ImmunoCAP > 0,35kU/L) were used as controls. In order to allow for simultaneous measurement of IgE responses, a number of purified proteins and protein mixes were used for analysis in a multiplexed capillary-flow based microarray assay.

## Results

Significant differences between children with symptoms and without symptoms were found for βconglycinin (Gly m 5) and glycinin (Gly m 6), the storage proteins of soybean. β-conglycinin showed higher response and glycinin, lower response by the tested patients.

## Conclusion

The highest and most prevalent IgE reacitivity in the group of children with soy allergy was directed to the two storage proteins of soy, β-conglycinin and glycinin. Both proteins appear to be good marker allergens for soy allergy in Brazilian children.

